# Acerola Cherry and Rosemary Extracts Improve Color and Delay Lipid Oxidation in Previously Frozen Beef

**DOI:** 10.3390/foods13101476

**Published:** 2024-05-10

**Authors:** Jessie B. Van Buren, Brooklyn Epperson, Sierra Jepsen, Mikayla Heimbuch, Kayleen Oliver, James Nasados, Phillip D. Bass, Michael J. Colle

**Affiliations:** Department of Animal, Veterinary, and Food Sciences, University of Idaho, Moscow, ID 83844, USA; jvanburen@uidaho.edu (J.B.V.B.); pbass@uidaho.edu (P.D.B.)

**Keywords:** antioxidants, frozen, shelf life, beef

## Abstract

Extending the shelf life of exported beef could increase international demand and producer profits. The objective was to evaluate the effects of topically applying combinations of acerola cherry powder and rosemary extract on the shelf life of frozen–thawed bone-in beef short rib and chuck roll steaks. Chuck rolls (IMPS 116A; *N* = 9) and bone-in short ribs (IMPS 123A; *N* = 18) were aged (7 d; 0 °C), frozen (30 d; −20 °C), and thawed (60–72 h; 0 °C). Steaks measuring 1.02 cm thick were treated and subjected to a 4 d retail display. Steaks were left untreated (control) or sprayed topically with acerola cherry powder (0.05%; A), rosemary extract (0.10%; R), or a combination (M1 = 0.05% A + 0.1% R; M2 = 0.1% A + 0.1% R; M3 = 0.05% A + 0.2% R; M4 = 0.1% A + 0.2% R). Chuck roll M2- and M4-treated steaks were redder than the control steaks on days 3 and 4 (*p* = 0.008), and antioxidant-treated steaks had less lipid oxidation on day 4 than the control steaks (*p* = 0.021). Bone marrow samples treated with R, M3, and M4 were redder than the control on days 1–3 (*p* = 0.014), and bone marrow treated with M3 was subjectively redder compared to the control on days 0 and 1 (*p* = 0.033). Topical antioxidants improve the redness and delay the oxidation of frozen–thawed beef.

## 1. Introduction

In 2022, U.S. beef and variety meat exports were valued at USD 11.68 billion and contributed USD 407.22 per steer or heifer processed [1]. South Korea exports grew and set a record, with exports valued at USD 2.7 billion, or USD 103.35 per fed head [1]. During extended shipping times from the U.S. to Korea, beef continues to age, potentially resulting in higher discoloration and oxidation rates of lean tissue and bone marrow [2,3,4,5,6]. The two specific cuts of beef that are of high importance are bone-in short ribs and chuck rolls because of the popularity of these two cuts in Korean cuisine [7].

By comparison, Australian beef has an advantage over U.S. beef in the Korean market because of the shorter transportation time and higher levels of vitamin E in the meat due to their feeding practices [8]. Researchers have seen benefits from including antioxidants in ground products [9,10,11,12] or on the surface of whole muscle steaks to extend their shelf life [13]. Van Buren et al. [14,15] recently found that in fresh, never-frozen beef, the topical application of acerola cherry powder and rosemary extract improved the marrow color in bone-in short ribs and surface discoloration in chuck rolls after 28 d of wet aging.

Beef subprimals can be frozen prior to shipping to prevent oxidation, which could be a potential solution during transport to improve shelf life [16]. However, freezing beef can shorten the shelf life of the product due to decreased redness and overall discoloration [17,18]. Potentially, beef could be frozen to limit the oxidation of subprimals during transport, and then, antioxidants could be topically applied to freshly cut steaks, limiting discoloration at the retail stage. 

Unfortunately, limited research has been conducted using antioxidants on beef following frozen storage and thawing. As previously stated, Van Buren et al. [14,15] found that acerola cherry powder and rosemary extract improve the shelf life of fresh beef. The present study aims to evaluate the effects of these antioxidants on previously frozen beef. Therefore, the objective of the current study was to evaluate the effect of the topical application of acerola cherry powder and rosemary extract in combination on the shelf life of previously frozen, bone-in beef short rib and chuck roll steaks, including the color, lipid oxidation, and fluid loss.

## 2. Materials and Methods

The materials and methods in this paper follow those of Van Buren et al. [14,15].

### 2.1. Product Preparation

From a commercial packing plant, USDA Choice beef chuck rolls (IMPS 116A; *N* = 9) and bone-in short ribs (IMPS 123A; *N* = 18) were purchased. The beef was transported under refrigeration (4 h; 4 °C) to the University of Idaho Meat Laboratory. The subprimals were wet aged at 0 °C for 7 d postfabrication followed by 30 d of frozen storage at −20 °C. The subprimals were thawed at 0 °C for 60–72 h prior to steak fabrication. Chuck rolls and short ribs, perpendicular to the bone, were cut into 1.02 cm thick steaks (*N* = 126 and *N* = 126, respectively). Steaks (n = 16 per treatment) were randomly assigned to a treatment group. The treatments included the following: untreated control (C), acerola cherry powder solution (0.05% A; Fortium A, Kemin Industries, Des Moines, IA), rosemary extract solution (0.10% R; Fortium R-WS 20, Kemin Industries), or a mixture of acerola cherry powder and rosemary extract (M1 = 0.05% A + 0.1% R; M2 = 0.1% A + 0.1% R; M3 = 0.05% A + 0.2% R; M4 = 0.1% A + 0.2% R). Antioxidant treatments were topically sprayed (2 mL) with a commercially available spray bottle (CleanCheck Commercial Sprayer, Tolco, Toledo, OH, USA).

The steaks were displayed in a retail display room (366 cm in length × 274 cm in width × 274 cm in height) at 2 °C for 4 d. The display room was equipped with natural white 4000 W lights, and the average light intensity was 849 lux (Fisherbrand Traceable Dual-Range Light Meter, Fisher Scientific, Waltham, MA, USA). To avoid potential effects due to the display location, the steaks were rotated in the retail environment daily.

### 2.2. Retail Fluid Loss

Immediately following the antioxidant treatment, the retail display steaks were weighed, placed on white foam trays (CKF Inc. #88142, Langley, BC, Canada), and overwrapped with an oxygen-permeable polyvinyl chloride film (oxygen transmission rate: 1450 cc/645 cm^2^ per 24 h; water vapor transmission rate: 17.0 g/645 cm^2^ per 24 h; Koch Industries, Inc., #7500-3815, Wichita, KS, USA). Following 4 d of retail display, the steaks were removed from the retail packaging and reweighed to determine the retail moisture loss. The percent retail fluid loss was calculated using the following equation:

$$\%\;Fluid\;Loss=\frac{Initial\;Weight-Final\;Weight}{Initial\;Weight}\times 100\%$$

### 2.3. Retail Color

The retail display steaks were allowed to bloom for at least 60 min, and then 2 objective color measurements per steak were obtained using a Nix Pro 2 Color Sensor (Nix Sensor Ltd., Hamilton, ON, Canada). Two objective color measurements were also taken for the bone marrow of the short rib steaks. The Nix Pro 2 Color Sensor was equipped with a 14 mm diameter measuring area and a 2° standard observer. The instrument was set to Illuminant D65 and Commission Internationale de l’Eclairage. L*, a*, and b* values were recorded.

Five trained evaluators measured the subjective color once daily. Each steak subjective color score was averaged across the evaluators prior to the analysis. The oxygenated lean color (1 = extremely bright cherry red, 8 = extremely dark red), discoloration (1 = none, 5 = extreme), surface discoloration (1 = no discoloration (0%), 6 = extensive discoloration (81% to 100%)), bone marrow color (1 = bright reddish pink to red, 7 = black), color uniformity (1 = uniform with no two-toning, 5 = extreme two-toning), and amount of browning (1 = no evidence of browning, 6 = dark brown) were measured following the American Meat Science Association guidelines [19].

### 2.4. Metmyoglobin-Reducing Activity

The nitric oxide metmyoglobin-reducing activity (MRA) was measured on the *Serratus ventralis* (SV) after the antioxidant treatment on day 0 and on day 4 following the protocols outlined in Section XI of the Meat Color Measurement Guidelines [19]. Two color measurements were obtained using a HunterLab MiniScan EZ (Reston, VA, USA) equipped with a 25-millimeter-diameter measuring area and a 10° standard observer. The instrument was set to Illuminant A, and the reflectance from 400 to 700 nm was recorded. Calibration occurred by measuring against black and white calibration tiles prior to measuring the color. The percentage of metmyoglobin (MMb) was calculated following the equations in Section XI of the Meat Color Measurements Guidelines [19]. The MRA was calculated as follows:

$$MRA=\left[\frac{Initial\;\%\;MMb-Final\;\%\;MMb}{Initial\;\%\;MMb}\right]\times 100$$

### 2.5. Oxygen Consumption

Oxygen consumption (OC) was measured on the SV after the antioxidant treatment on day 0 following the protocols outlined in the Meat Color Measurement Guidelines [14]. Color measurements were taken similar to the MRA protocol. The percentage of oxymyoglobin (OMb) was calculated following the equations in Section XI of the Meat Color Measurement Guidelines [19]. The OC was calculated as follows:

$$OC=\left[\frac{Initial\;\%\;OMb-Final\;\%\;OMb}{Initial\;\%\;OMb}\right]\times 100$$

### 2.6. Lipid Oxidation

Thiobarbituric acid reactive substances (TBARSs) were analyzed in duplicate on day 0 after treatment and on day 4 of retail display following the protocol in Section XI, Appendix O of the Meat Color Measurement Guidelines [19]. The samples weighed 1 g and were cut from the SV, avoiding the steak edge, large pieces of fat, and connective tissue.

### 2.7. Statistical Analysis

All statistical analyses were conducted using SAS V 9.4 (SAS Inc., Cary, NC, USA). The data were analyzed using a mixed model analysis of variance. The antioxidant treatments, retail display times, and their interactions were assumed as fixed effects. The analyses performed at more than one time point (MRA, color, and lipid oxidation) had a split-plot design with repeated measures. The retail display time was considered a repeated measure modeled as a compound symmetric correlation structure. The treatment least squares mean differences were assessed through pair-wise comparisons for significant effects. The significance was determined at *p* < 0.05.

## 3. Results and Discussion

### 3.1. Retail Fluid Loss

Antioxidant treatments did not impact fluid loss during retail display for the chuck roll (*p* = 0.827; Table 1) or bone-in short rib steaks (*p* = 0.142; Table 2). Chuck roll steaks lost an average of 1.12% of fluid, and short rib steaks lost an average of 1.68% of fluid. Typically, freezing beef results in a greater purge loss during storage and thawing [20,21,22]. However, both subprimal averages were below the limit (2%) to be considered acceptable fluid loss and were below the threshold (4%) for causing a loss in profits [23]. The low retail fluid loss may be due to the loss of fluid in the packaging during thawing. Future research should include purge loss during frozen storage and thawing.

### 3.2. Retail Objective Color

In the chuck roll steaks, there was an interaction between the day of retail display and antioxidant treatment for mean a* values (*p* = 0.008; Table 3). The treatments did not differ on days 0, 1, or 2. On day 3, the steaks treated with M2 or M4 were redder than the control steaks or steaks only treated with A. On day 4, the steaks treated with R, M1, M2, and M4 were redder than the control steaks or steaks treated with A. At low concentrations, ascorbic acid, the active ingredient in A, can act as a pro-oxidant, which may result in less redness [9,24]. Additionally, from day 3 to day 4, there were large declines in the steaks treated with A and the control steaks. These were the only steaks to drop below the consumer redness (a*) acceptance threshold of 14.5 [25]. Previously frozen beef has been found to discolor or lose redness (a*) quicker than never-frozen beef [17,18]. However, when A and R were used in combination, the ascorbic acid was able to regenerate the tocopherols to delay myoglobin oxidation and discoloration [26]. An interaction was not observed for mean values of L* or b* (*p* = 0.337 and *p* = 0.555, respectively). Additionally, differences were not observed between the antioxidant treatments for mean values of L* or b* (*p* = 0.375 and *p* = 0.676, respectively; Table 1).

In the lean tissue of the short ribs, interactions between the antioxidant treatment and day of retail display were not observed for mean values of L*, a*, or b* (*p* = 0.337, *p* = 0.125, and *p* = 0.585, respectively). Additionally, differences between the antioxidant treatments were not observed for mean values of L*, a*, or b* (*p* = 0.105, *p* = 0.293, and *p* = 0.824, respectively; Table 2). Previous research also did not show differences in the objective color measurements of short rib steaks between antioxidant treatments [14]. Objective measurements were taken on the SV for both chuck roll and short rib steaks, which has previously been found to have low color stability [27]. The increased color stability in the short rib steaks may be due to decreased use of this area during locomotion on the live animal.

An interaction for a* was observed in the bone marrow of the short rib steaks between the day of retail display and antioxidant treatment (*p* = 0.014; Table 4). Interestingly, the control bone marrow had a sharp decline in redness (a*) from day 0 to day 1, whereas all antioxidant-treated bone marrow samples had a delayed or more gradual decline in redness, and all antioxidant-treated bone marrow samples were redder than the control on day 1. Previous research had shown that antioxidants were effective in improving the bone marrow color by delaying hemoglobin oxidation and myoglobin oxidation similarly [2,14,15,28,29]. Bone marrow samples treated with R, M3, and M4 were also, on average, redder than the control on days 2 and 3. These results dispute previous knowledge that lipid soluble antioxidants, such as rosemary extract in this study, do not inhibit discoloration in bone marrow [30]. An interaction was not observed for L* or b* (*p* = 0.366 and *p* = 0.055, respectively). The control bone marrow had a lower mean b* value than any of the antioxidant-treated bone marrow samples (*p* = 0.001; Table 2). The L* values were not influenced by antioxidant treatments (*p* = 0.243; Table 2).

### 3.3. Retail Subjective Color

In the chuck roll steaks, there were interactions between the day of retail display and antioxidant treatment for the amount of browning, discoloration, and color uniformity (*p* = 0.015, *p* = 0.001, and *p* = 0.003, respectively; Table 3). On days 0 and 1, the treatments did not differ in the amount of browning, discoloration, or color uniformity. On day 2, the steaks treated with M4 had less browning than the control steaks. On day 3, the steaks treated with R or a mixture had less browning than the control steaks or steaks treated with A. Similar results were seen on day 4, with the exception of M1, which had similar browning to the control and A steaks. All treatments increased in browning each day of retail display except for the steaks treated with M4, which had similar amounts of browning on days 1 and 2. Starting on day 2, the steaks treated with a mixture had less discoloration than the control steaks. Similar results were seen on day 3, with the addition of steaks treated with R having less discoloration than the control steaks. On day 4, the steaks treated with R, M1, M3, and M4 had less discoloration than the control steaks or steaks treated with A. Consumers use color as an indicator of freshness [30] and prefer bright cherry-red meat [31]. By delaying discoloration, the meat industry can decrease food waste and improve the sustainability of beef [32]. The steaks treated with M2 and M4 were more uniform in color than the control steaks on day 2. The following day, the steaks treated with R, M2, and M3 were more uniform than the control steaks. Lastly, on day 4, similar to amount of browning and discoloration results, the steaks treated with R or a mixture were more uniform than the control steaks. Chuck roll steaks contain multiple muscles with varying degrees of color stability [27]. Utilizing antioxidants topically on other multi-muscle cuts may further improve the uniformity and consumer desirability at the retail level. There was no interaction for oxygenated lean color or surface discoloration (*p* = 0.064 and *p* = 0.096, respectively). Additionally, the antioxidant treatment did not impact the oxygenated lean color or surface discoloration (*p* = 0.676 and *p* = 0.462, respectively; Table 1).

In the short ribs, an interaction between the antioxidant treatment and day of retail display was only observed for the bone marrow color (*p* = 0.033; Table 4). Differences between the treatments started on day 0, with marrow treated with A, M2, and M3 being redder than the control marrow. On day 1, only marrow treated with M3 was redder than the control marrow. On days 2, 3, and 4, none of the treatments differed from the control marrow. While differences were seen later in the retail display between the antioxidant treatments in the objective redness, similar results were not seen subjectively. Previous research has shown rapid discoloration, or graying, of bone marrow and presented limited options to mitigate the discoloration, such as packaging without oxygen [33]. Continued research is needed to further delay the discoloration of bone marrow. An interaction was not observed for the oxygenated lean color, amount of browning, discoloration, surface discoloration, or color uniformity (*p* = 0.905, *p* = 0.896, *p* = 0.569, *p* = 0.763, and *p* = 0.833, respectively). Additionally, the antioxidant treatment did not impact the oxygenated lean color, amount of browning, discoloration, surface discoloration, or color uniformity (*p* = 0.215, *p* = 0.725, *p* = 0.786, *p* = 0.656, and *p* = 0.858, respectively; Table 2).

### 3.4. Metmyoglobin-Reducing Activity

An interaction between the day of retail display and antioxidant treatment was not observed for MRA in the chuck roll or short rib steaks (*p* = 0.315 and *p* = 0.580, respectively). Additionally, the MRA did not differ between antioxidant treatments in chuck roll (*p* = 0.165; Table 1) or short rib (*p* = 0.438; Table 2) steaks. Previous research has indicated that MRA is limited by the availability of NADH and is not influenced by the presence of antioxidants [34].

### 3.5. Oxygen Consumption

The oxygen consumption rate did not differ between treatments in the chuck roll steaks (*p* = 0.553; Table 1), similar to previous research [13,14]. However, the oxygen consumption rate was greatly decreased in short rib steaks treated with R or a mixture (*p* = 0.001; Table 2). Moderate oxygen consumption could lengthen the shelf life by regenerating NADH to be used for MRA [35].

### 3.6. Lipid Oxidation

In the chuck roll steaks, an interaction was observed between the antioxidant treatment and day of retail display for lipid oxidation (*p* = 0.021; Table 3). All treatments increased from day 0 to day 4, as expected [10,13]. On day 0, there was no difference between the antioxidant treatments, but on day 4, all antioxidant-treated steaks had less lipid oxidation than the control steaks. Additionally, the steaks treated with a mixture of the antioxidants had less oxidation than the steaks treated with A. Previous strategies have incorporated antioxidants into the feed for live cattle or into ground meat products to delay lipid oxidation [8,9,10,11,12]. By applying antioxidants topically, the meat industry can delay lipid oxidation in high-value cuts and be competitive in international markets without adjusting U.S. feeding strategies. On day 4, the steaks treated with A as well as the control steaks surpassed the threshold for consumers to detect off-flavors (TBARSs > 1.0) [36,37]. Ascorbic acid in A is more commonly used as a chelator to delay myoglobin oxidation, whereas tocopherols in R are more active in delaying lipid oxidation by acting as a radical quencher [26]. Therefore, it is not surprising that antioxidant treatments containing R outperformed the steaks treated with A and the control steaks.

In the short rib steaks, an interaction was observed between the antioxidant treatment and day of retail display for lipid oxidation (*p* = 0.028; Table 4). The control steaks as well as the steaks treated with A, R, and M2 increased in oxidation from day 0 to 4, whereas the steaks treated with M1, M3, and M4 did not significantly increase. On day 0, there was no difference between the antioxidant treatments. On day 4, the control steaks and steaks treated with R had the highest level of oxidation. The steaks treated with A or a mixture had less oxidation than the control steaks. These results contradict the results found in the chuck rolls, where R performed better than A, but ultimately, none of the treatments on day 0 or day 4 came near the threshold for consumers to detect off-flavors (TBARSs > 1.0) [36,37]. In future research, extending the retail display time may help determine how many days are needed for the control steaks to reach the threshold. Similar to the color results, it is interesting to note that all TBARS samples were taken from the SV (low color stability [27]) in the short rib and chuck roll steaks. However, the oxidation values within chuck roll steaks are noticeably higher than in the short rib steaks on day 4. Again, this may be due to the difference in the functionality of the muscle at the different anatomical locations.

## 4. Conclusions

Limited research had been conducted previously that looked at applying antioxidants to frozen–thawed beef to extend the retail shelf life. With the results from this study, it is recommended that retailers apply M3 (0.05% acerola cherry powder and 0.2% rosemary extract) to frozen–thawed bone-in short rib steaks to improve the redness of the bone marrow and delay lipid oxidation. Additionally, retailers should apply M4 (0.10% acerola cherry powder and 0.2% rosemary extract) to frozen–thawed chuck roll steaks to delay lipid oxidation, browning, discoloration, and two-toning, as well as improve redness. Applying acerola cherry powder and rosemary extract to other cuts with low color stability or to bone-in steaks may extend the shelf life of additional frozen–thawed beef products.

## Figures and Tables

**Table 1:** Estimated mean effects of topical antioxidant treatment on chuck roll steak fluid loss, color, metmyoglobin-reducing activity, oxygen consumption, and lipid oxidation (*N* = 63).

Trait		Topical Antioxidant Treatment ^1^							
	Control	A	R	M1	M2	M3	M4	*p*-Value	SEM
Retail fluid loss, %	1.07	1.12	1.19	1.06	1.14	1.24	1.06	0.823	0.10
L*	33.7	34.0	35.4	38.8	35.0	39.8	39.5	0.375	2.6
b*	15.3	13.4	14.2	13.9	14.2	14.3	14.5	0.676	0.7
Oxygenated lean color ^2^	4.2	4.4	4.1	4.0	4.0	4.0	4.0	0.676	0.2
Surface discoloration ^3^	2.6	2.7	2.4	2.3	2.3	2.4	2.4	0.462	0.1
Metmyoglobin-reducing activity, %	8.07	8.54	6.85	10.33	8.96	11.09	11.91	0.165	1.77
Oxygen consumption, %	49.93	44.84	42.42	36.90	45.57	43.47	41.64	0.553	4.65

^1^ Treatments included an untreated control, topically sprayed (2 mL) with a 0.05% acerola cherry powder solution (A), topically sprayed (2 mL) with a 0.10% rosemary extract solution (R), or topically sprayed (2 mL) with a mixture of the acerola cherry powder and rosemary extract (M1 = 0.05% A + 0.1% R; M2 = 0.1% A + 0.1% R; M3 = 0.05% A + 0.2% R; M4 = 0.1% A + 0.2% R). ^2^ Oxygenated lean color scale: 1 = extremely bright cherry red; 8 = extremely dark red. ^3^ Surface discoloration scale: 1 = no discoloration (0%); 6 = extensive discoloration (81–100%).

**Table 2:** Estimated mean effects of topical antioxidant treatment on bone-in short rib steak fluid loss, color, metmyoglobin-reducing activity, and oxygen consumption (*N* = 63).

Trait		Topical Antioxidant Treatment ^1^							
	Control	A	R	M1	M2	M3	M4	*p*-Value	SEM
Retail fluid loss, %	1.65	1.77	1.69	1.74	1.67	1.61	1.63	0.142	0.05
Bone marrow L*	41.7	47.5	41.0	38.4	43.3	43.5	40.0	0.243	2.5
Bone marrow b*	9.1 ^b^	12.3 ^a^	13.4 ^a^	12.1 ^a^	12.2 ^a^	12.8 ^a^	12.8 ^a^	0.001	0.7
Lean L*	37.6	3.3	40.5	40.2	40.5	41.8	40.2	0.105	1.0
Lean a*	19.2	18.7	18.2	18.6	18.8	17.3	17.4	0.293	0.6
Lean b*	12.7	13.5	13.7	13.7	13.6	13.6	13.3	0.824	0.5
Oxygenated lean color ^2^	5.5	5.2	5.2	5.3	5.2	5.2	5.4	0.940	0.2
Amount of browning ^3^	2.3	2.1	2.2	2.2	2.3	2.0	2.4	0.725	0.2
Discoloration ^4^	2.0	1.9	1.9	2.0	2.0	1.9	2.2	0.786	0.1
Surface discoloration ^5^	2.2	2.0	2.2	2.2	2.2	2.0	2.5	0.656	0.2
Color uniformity ^6^	2.0	1.8	1.9	1.8	1.8	1.8	1.9	0.858	0.1
Metmyoglobin-reducing activity, %	12.53	14.30	11.92	11.52	11.89	12.88	14.05	0.438	1.09
Oxygen consumption, %	54.58 ^a^	56.65 ^a^	46.00 ^b^	43.37 ^b^	40.82 ^b^	39.70 ^b^	40.15 ^b^	0.001	3.19

^ab^ Within a row, means without a common superscript differ (*p* < 0.05). ^1^ Treatments included an untreated control (C), topically sprayed (2 mL) with a 0.05% acerola cherry powder solution (A), topically sprayed (2 mL) with a 0.10% rosemary extract solution (R), or topically sprayed (2 mL) with a mixture of the acerola cherry powder and rosemary extract (M1 = 0.05% A + 0.1% R; M2 = 0.1% A + 0.1% R; M3 = 0.05% A + 0.2% R; M4 = 0.1% A + 0.2% R). ^2^ Oxygenated lean color scale: 1 = extremely bright cherry red; 8 = extremely dark red. ^3^ Amount of browning scale: 1 = no evidence of browning; 6 = dark brown. ^4^ Discoloration scale: 1 = none; 5 = extreme. ^5^ Surface discoloration scale: 1 = no discoloration (0%); 6 = extensive discoloration (81–100%). ^6^ Color uniformity scale: 1 = uniform with no two-toning; 5 = extreme two-toning.

**Table 3:** Estimated mean effects of topical antioxidant treatment and retail display time on chuck roll steak color and lipid oxidation (*N* = 63).

Trait	Day of Display	Topical Antioxidant Treatment ^1^								
		Control	A	R	M1	M2	M3	M4	*p*-Value	SEM
a* ^2^	0	20.0 ^w^	21.0 ^w^	20.1 ^w^	20.7 ^w^	21.0 ^w^	20.0 ^w^	20.3 ^w^	0.008	0.7
	1	18.3 ^wx^	18.6 ^x^	18.5 ^wx^	19.1 ^wx^	18.4 ^x^	18.5 ^wx^	19.8 ^wx^		
	2	17.7 ^xy^	16.6 ^y^	17.1 ^x^	17.6 ^xy^	18.0 ^xy^	16.9 ^xy^	17.9 ^y^		
	3	15.9 ^bc,y^	15.8 ^c,y^	17.7 ^abc,x^	17.9 ^ab,xy^	18.0 ^a,xy^	16.7 ^abc,xy^	18.3 ^a,xy^		
	4	14.0 ^c,z^	11.9 ^d,z^	17.8 ^a,x^	16.2 ^ab,y^	16.3 ^ab,y^	15.2 ^bc,y^	16.8 ^ab,y^		
Amount of browning ^3^	0	1.0 ^z^	1.1 ^z^	1.0 ^z^	1.1 ^z^	1.0 ^z^	1.0 ^z^	1.0 ^y^	0.015	0.2
	1	1.8 ^y^	1.9 ^y^	1.6 ^y^	1.8 ^y^	1.7 ^y^	1.6 ^y^	1.8 ^x^		
	2	2.6 ^a,x^	2.4 ^ab,x^	2.2 ^ab,x^	2.2 ^ab,x^	2.3 ^ab,x^	2.2 ^ab,x^	2.1 ^b,x^		
	3	3.7 ^a,w^	3.7 ^a,w^	2.9 ^b,w^	2.8 ^b,w^	3.0 ^b,w^	3.1 ^b,w^	3.0 ^b,w^		
	4	4.5 ^a,v^	4.4 ^a,v^	3.8 ^bc,v^	4.0 ^ab,v^	3.8 ^bc,v^	3.5 ^c,v^	3.6 ^bc,v^		
Discoloration ^4^	0	1.2 ^z^	1.2 ^z^	1.2 ^y^	1.2 ^y^	1.3 ^y^	1.1 ^y^	1.3 ^y^	0.001	0.2
	1	1.9 ^y^	1.7 ^y^	1.7 ^x^	1.7 ^x^	1.7 ^x^	1.6 ^x^	1.7 ^x^		
	2	2.5 ^a,x^	2.2 ^ab,x^	2.1 ^ab,w^	2.0 ^b,x^	1.9 ^b,x^	1.8 ^b,x^	1.9 ^b,x^		
	3	3.4 ^a,w^	3.5 ^a,w^	2.9 ^b,v^	2.8 ^b,w^	2.7 ^b,w^	2.9 ^b,w^	2.8 ^b,w^		
	4	4.1 ^a,v^	4.1 ^a,v^	3.2 ^c,v^	3.6 ^bc,v^	3.7 ^ab,v^	3.3 ^c,v^	3.4 ^bc,v^		
Color uniformity ^5^	0	1.3 ^z^	1.3 ^y^	1.4 ^x^	1.6 ^y^	1.3 ^y^	1.4 ^y^	1.4 ^y^	0.003	0.1
	1	1.8 ^y^	1.8 ^x^	1.7 ^x^	1.9 ^xy^	1.9 ^x^	2.0 ^x^	1.9 ^x^		
	2	2.4 ^a,x^	2.0 ^ab,x^	2.3 ^a,w^	2.1 ^ab,x^	1.9 ^b,x^	2.0 ^ab,x^	1.9 ^b,x^		
	3	2.9 ^a,w^	2.9 ^ab,w^	2.4 ^c,w^	2.7 ^abc,w^	2.4 ^c,w^	2.5 ^bc,w^	2.6 ^abc,w^		
	4	3.6 ^a,v^	3.3 ^ab,v^	3.2 ^b,v^	3.2 ^bc,v^	3.2 ^bc,v^	2.9 ^c,v^	3.1 ^bc,v^		
Lipid oxidation ^6^	0	0.29 ^z^	0.23 ^z^	0.15 ^z^	0.19 ^z^	0.15 ^z^	0.14 ^z^	0.17 ^z^	0.021	0.10
	4	1.40 ^a,y^	1.08 ^b,y^	0.85 ^bc,y^	0.77 ^c,y^	0.68 ^c,y^	0.66 ^c,y^	0.61 ^c,y^		

^abcd^ Within a trait and day, means without a common superscript differ (*p* < 0.05). ^vwxyz^ Within a trait and treatment, means without a common superscript differ (*p* < 0.05). ^1^ Treatments included an untreated control (C), topically sprayed (2 mL) with a 0.05% acerola cherry powder solution (A), topically sprayed (2 mL) with a 0.10% rosemary extract solution (R), or topically sprayed (2 mL) with a mixture of the acerola cherry powder and rosemary extract (M1 = 0.05% A + 0.1% R; M2 = 0.1% A + 0.1% R; M3 = 0.05% A + 0.2% R; M4 = 0.1% A + 0.2% R). ^2^ −60 (green) to +60 (red) ^3^ Amount of browning scale: 1 = no evidence of browning; 6 = dark brown. ^4^ Discoloration scale: 1 = none; 5 = extreme. ^5^ Color uniformity scale: 1 = uniform with no two-toning; 5 = extreme two-toning. ^6^ mg malondialdehyde/kg meat.

**Table 4:** Estimated mean effects of topical antioxidant treatment and retail display time on bone-in short rib steak color and lipid oxidation (*N* = 63).

Trait	Day of Display	Topical Antioxidant Treatment ^1^								
		Control	A	R	M1	M2	M3	M4	*p*-Value	SEM
Bone marrow a* ^2^	0	16.0 ^bc,w^	15.7 ^c,w^	20.1 ^ab,w^	21.1 ^a,w^	17.6 ^bc,w^	17.1 ^bc,w^	19.2 ^ab,w^	0.014	1.1
	1	10.4 ^c,x^	13.0 ^b,x^	18.0 ^a,w^	18.2 ^a,x^	16.9 ^a,w^	16.8 ^a,w^	16.3 ^a,x^		
	2	10.3 ^d,x^	11.7 ^cd,xy^	17.9 ^a,w^	14.4 ^bc,y^	12.9 ^bcd,x^	15.7 ^ab,wx^	14.8 ^ab,xy^		
	3	10.5 ^b,x^	9.3 ^b,yz^	14.3 ^a,x^	12.3 ^ab,y^	13.7 ^a,x^	13.7 ^a,x^	14.0 ^a,xy^		
	4	10.8 ^ab,x^	9.2 ^b,z^	12.4 ^a,x^	12.8 ^a,y^	13.4 ^a,x^	13.3 ^a,x^	12.5 ^a,y^		
Bone marrow color ^3^	0	4.9 ^a,z^	3.6 ^b,z^	4.1 ^ab,z^	4.2 ^ab,z^	3.4 ^b,z^	3.7 ^b,z^	4.1 ^ab,z^	0.033	0.3
	1	5.6 ^a,y^	4.9 ^ab,y^	4.8 ^ab,y^	5.5 ^a,y^	4.7 ^ab,y^	4.5 ^b,y^	5.3 ^ab,y^		
	2	6.1 ^ab,x^	5.5 ^ab,x^	5.7 ^ab,x^	6.3 ^a,x^	5.7 ^ab,x^	5.3 ^b,x^	6.2 ^ab,x^		
	3	6.4 ^ab,wx^	5.9 ^ab,wx^	6.2 ^ab,w^	6.7 ^a,wx^	6.1 ^ab,w^	5.8 ^b,w^	6.7 ^a,w^		
	4	6.7 ^ab,w^	6.0 ^b,w^	6.4 ^ab,w^	6.9 ^a,w^	6.1 ^ab,w^	6.0 ^ab,w^	6.7 ^ab,w^		
Lipid oxidation ^4^	0	0.09 ^z^	0.09 ^z^	0.06 ^z^	0.10 ^z^	0.09 ^z^	0.10 ^z^	0.12 ^z^	0.028	0.05
	4	0.33 ^a,y^	0.22 ^bc,y^	0.27 ^ab,y^	0.16 ^c,z^	0.20 ^bc,y^	0.17 ^c,z^	0.22 ^bc,z^		

^abcd^ Within a trait and day, means without a common superscript differ (*p* < 0.05). ^wxyz^ Within a trait and treatment, means without a common superscript differ (*p* < 0.05). ^1^ Treatments included an untreated control (C), topically sprayed (2 mL) with a 0.05% acerola cherry powder solution (A), topically sprayed (2 mL) with a 0.10% rosemary extract solution (R), or topically sprayed (2 mL) with a mixture of the acerola cherry powder and rosemary extract (M1 = 0.05% A + 0.1% R; M2 = 0.1% A + 0.1% R; M3 = 0.05% A + 0.2% R; M4 = 0.1% A + 0.2% R). ^2^ −60 (green) to +60 (red) ^3^ Bone marrow color scale: 1 = bright reddish pink to red; 7 = black. ^4^ mg malondialdehyde/kg meat.

## Data Availability

The original contributions presented in the study are included in the article, further inquiries can be directed to the corresponding author.
